# FDG PET/CT as a survival prognostic factor in patients with advanced renal cell carcinoma

**DOI:** 10.1007/s10238-018-0539-9

**Published:** 2018-11-28

**Authors:** Violetta Pankowska, Bogdan Malkowski, Mateusz Wedrowski, Ewelina Wedrowska, Krzysztof Roszkowski

**Affiliations:** 1 Nuclear Medicine Department, Oncology Centre, Bydgoszcz, Poland; 2 Department of Positron Emission Tomography and Molecular Diagnostic, Nicolaus Copernicus University, Collegium Medicum, Bydgoszcz, Poland; 3 Department of Gene Therapy, Faculty of Medicine, Nicolaus Copernicus University, Collegium Medicum, Bydgoszcz, Poland; 4 Department of Oncology, Radiotherapy and Gynecologic Oncology, Faculty of Health Sciences, Nicolaus Copernicus University, Collegium Medicum, Bydgoszcz, Poland

**Keywords:** Renal cell carcinoma, FDG PET/CT, Prognosis, Targeted molecular therapy

## Abstract

Accurate prediction of the outcome of molecular target-based treatment in advanced renal cell carcinoma (RCC) is an important clinical problem. Positron emission tomography/computed tomography using [18F]-2-fluoro-2-deoxyglucose (FDG PET/CT) is a noninvasive tool for the assessment of glucose accumulation which can be a marker of the biological characteristics of the tumor. In this paper, we assess FDG PET/CT as a survival prognostic marker in patients with advanced RCC. The study included 121 patients treated in the years 2011–2016 with a diagnosis of advanced renal cell carcinoma (stage IV, multifocal metastases in all patients). Assessment using FDG PET/CT was conducted by measuring the maximum standard uptake value (SUVmax) for the marker used (the highest SUV measurement result for each patient in a single examination). SUVmax measurements were compared with various clinical risk factors used as prognostic markers. The median follow-up period was 19 months (ranging from 3 to 61 months). SUVmax measurements in all patients ranged from 1.3 to 30.0 (median 6.9). Higher SUVmax was correlated with poorer prognosis. Multi-way analysis with standard risk factors revealed that SUVmax was an independent factor for overall survival (OS; *p* < 0.003, hazard ratio 1.312, 95% CI 1.147–1.346). For SUVmax < 7.0, median OS was 32 months. For 7.0 ≤ SUVmax < 12.0, median OS was 12.5 months. For SUVmax ≥ 12.0, median OS was 10 months. The differences were statistically significant. A preliminary SUVmax assessment conducted using FDG PET/CT can provide information useful in the prediction of survival of patients with advanced RCC.

## Introduction

Renal cell carcinoma (RCC) is not a common cancer, constituting 3% of all tumors in adults [1]. Metastases are detected in approximately 30% of RCC patients, with further 30–40% developing metastases after radical nephrectomy with curative intent [2, 3]. Cytokine treatments have been available for advanced RCC patients for a long time and have been associated with an uncertain prognosis [4, 5]. Molecular targets, such as vascular endothelial growth factor (VEGF) or mTOR kinase, have been selected for newer therapeutics to improve the therapeutic index [6–9] and are recommended as the main treatments for advanced RCC [10, 11]. It is commonly known that prognosis in RCC patients can vary, and the guidelines recommend treatments employing prognostic classifications based on a combination of clinical information and laboratory data [8, 10, 11].

Positron emission tomography/computed tomography using [18F]-2-fluoro-2-deoxyglucose (FDG PET/CT) is a useful noninvasive tool for the assessment of glucose metabolism which can be a marker of the biological activity of the tumor. We focused on the standard uptake value (SUV). SUVmax was described by other authors as a simplified quantitative measure of FDG accumulation (i.e., the highest SUV of all RCC lesions in each patient) that predicted overall survival (OS) of patients with advanced RCC [12].

In this article, we present our analyses of OS of patients with SUVmax higher and lower than the threshold value of 7.8. Kayani reported that high SUVmax was correlated with a shorter OS in patients treated with the tyrosine kinase inhibitor (TKI) sunitinib [13]. In another study, Chen reported that the baseline SUVmax was correlated with the OS of RCC patients treated with everolimus, an oral mTOR inhibitor (mTORi) [14]. Other authors also supported the usefulness of FDG PET/CT as a prognostic tool for patients with RCC [15, 16].

In this study, we present our results from a longer follow-up period.

## Methods

### Patients

The analysis was conducted in 121 patients diagnosed with clear-cell RCC, treated at the Oncology Center in Bydgoszcz, Poland, between April 2011 and April 2016.

### Imaging

The patients reported for examination at least 6 h after the last meal and drank 0.5–0.75 L of still unflavored mineral water.

After administration of the radiopharmaceutical FDG, the patients waited for the examination for approximately 1 h in a recumbent or seated position and were advised to reduce physical activity. During that time, physiological distribution of the marker in the body occurred. After intravenous administration, most of the FDG is quickly cleared from the vascular space (T_1/2_ under 1 min) and undergoes biodistribution in tissues (T_1/2_ up to 1.5 h). Approximately 4% of the administered activity accumulates in the heart, 8% in the brain, 5% in the liver and 3% in the lungs. The remaining 80% of the administered activity is distributed in all other tissues. Approximately 30% of the fraction distributed in all other tissues (24% of the total administered activity) is excreted with urine (T_1/2_ 12 min—25% and T_1/2_ 1.5 h—75%). Physiological uptake of FDG by brown adipose tissue has also been observed.

*Isotope* Fluorine-18—positron-emitting isotope with a half-life of 109.771 min.

To conduct the examination, FDG with an activity of 5–7 MBq/kg b.w. was administered. Directly after the administration of FDG, 20 mg of furosemide was (optionally) administered to increase renal excretion.

- *Acquisition start time* 60 minutes after FDG administration.
- *Patient body position* Lying in a supine position, with hands behind the head.
- *Imaging range* from the cranial base to mid-thigh. In cases requiring additional acquisitions: areas of the head and limbs.
- The examinations were conducted using a PET/CT scanner Biograph mCT 128 (SIEMENS).
- *Acquisition parameters* CT WB: Topogram—Standard, Eff.mAs—Care Dose 4d, kV—120, Slice—5.0 mm, Acq—32×1.2 mm, Pitch—0.7, Direction—Craniocaudal, Kernel—B30f, FoV—780 mm, Increment—3.0 mm.
- *PET WB* Isotope—F-18, Pharm—FDG, Scan Range—Match CT Range, Scan Duration/Bed—1.8 min.
- *PET recon 1* Output Image—Corrected, Recon Meth—TRUeX+TOF(UltraHD-PET), Iteration—2, Subset—21, Image Size—200, Filter—Gaussian, Zoom—1.0, FWHM—5.0.
- *PET recon 2* Output Image—Uncorrected, Recon Meth—Iterative+TOF, Iteration—3, Subset—21, Image Size—200, Filter—Gaussian, Zoom—1.0, FWHM—5.0.
- CT was performed as per protocol following diagnostic criteria, without or with intravenous administration of contrast depending on the clinical requirements.
- *Image reconstruction* Iterative reconstruction algorithm taking into account the correction of absorption and dispersion phenomena.
- *Processing of quantitative and image data* Assessment of the intensity of distribution of the marker in pathological lesions (SUV, semiquantitative assessment).

### Statistical analysis

The arithmetic mean and median OS were calculated using the Microsoft® Excel 2011 spreadsheet. Statistical significance of differences between the obtained results was checked using the logrank test (*p* < 0.05) in the Statistica software v. 13.0 by StatSoft.

OS was calculated between the date of the FDG PET/CT examination and the date of death. OS curves were estimated using the Kaplan–Meier method and compared using the logrank test. The impact of SUVmax and other standard clinicopathological factors (fitness, gap between diagnosis and start of treatment, LDH and calcium levels, age, sex and histopathological assessment) on OS were analyzed using monodimensional Cox regression model at *p* < 0.05 and using a multidimensional Cox model.

**Fig. 1:**
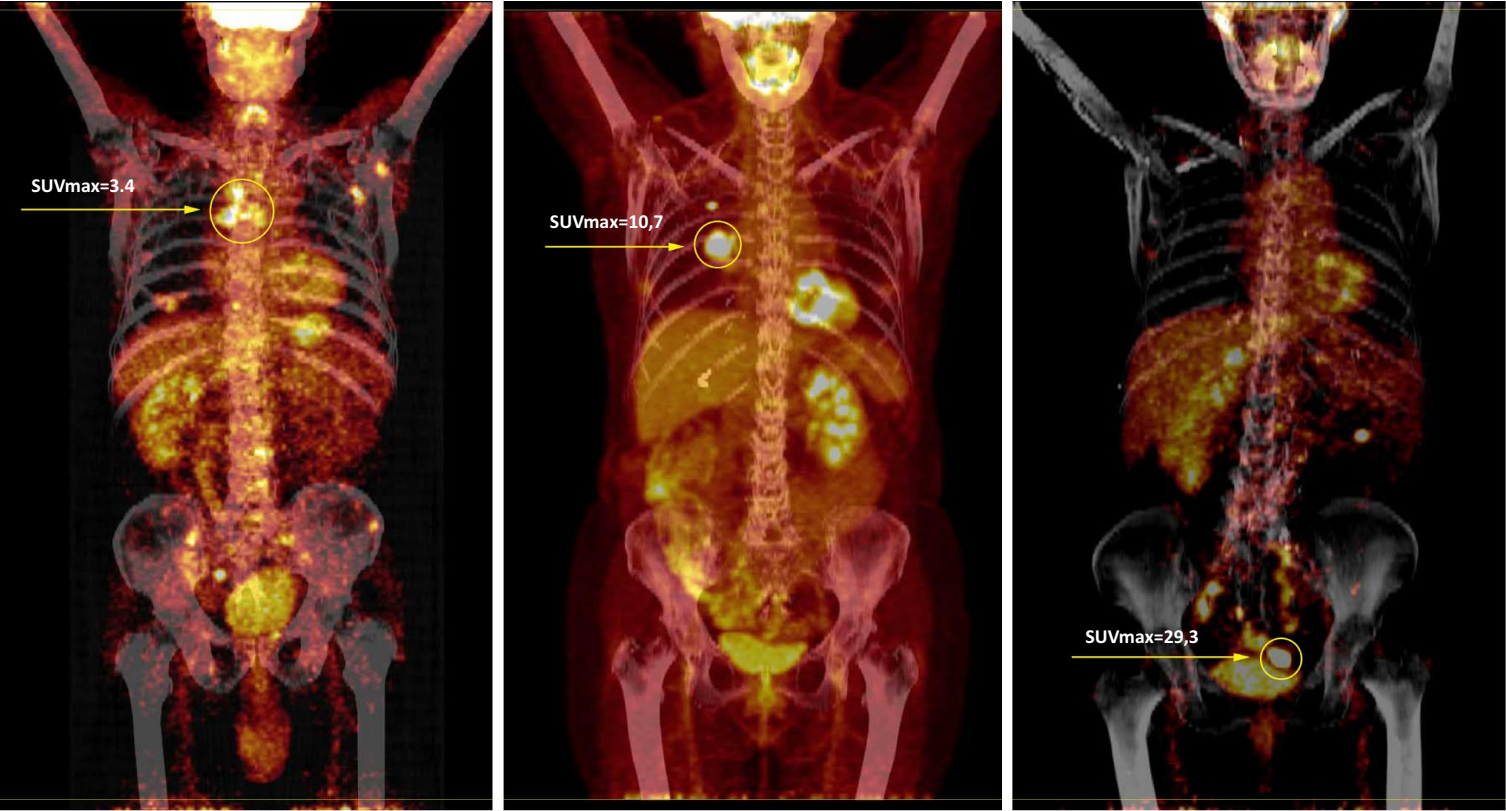
Sample FDG PET/CT images as a function of identified SUVmax cutoff points

Sample FDG PET/CT images as a function of identified SUVmax cutoff points (Fig. 1).

## Results

### Patient characteristics

The clinical characteristics of the 121 patients are presented in Table 1. Among 79 patients with stage IV RCC, 42 had not previously undergone nephrectomy. FDG PET/CT assessment in 12 patients who had received prior treatment was conducted more than 3 weeks after the end of the previous treatment.

**Table 1:** Characteristic patients

Characteristic	No. of patients (%)
No. of patients	121
Sex	
Male	94
Female	27
Age, years	
Median (range)	64 (41–83)
Pathology	
Clear cell	91
Papillary	19
Hemodialysis	4
Unclassified	7
Prior nephrectomy	
Yes	79
No	42
Disease status	
Recurrent	72
Metastatic	63
Regional	9
Stage IV	49
Metastatic	46
Locoregional	3
Prior systematic therapy	
Yes	12
IFN-α	7
IFN-α/sorafenib	2
Sorafenib	2
Sunitinib	1

*IFN*-*α* interferon-α

### Treatment methods

The median follow-up period was 19 months (ranging from 6 to 61 months). During the follow-up, 60 patients were treated with a single intervention (20 with sorafenib, 31 with sunitinib), 29 were treated with two interventions (20 with TKI and mTORi, 9 with two TKIs), while the remaining patients were treated with three or more interventions (Table 2). Sixty-three tumor-related deaths were observed; the remaining 58 patients were confirmed to be alive at the time of writing of this paper. There were deaths due to other causes.

**Table 2:** Interventions after PET/CT evaluation

Interventions	No. of patients	
Single intervention	60	
Sunitinib		31
Sorafenib		20
IFN-α		9
2 interventions	29	
TKI to mTORI		20
TKI to TKI		9
3 = < interventions	32	

*IFN*-*α* interferon-α, *TKI* tyrosine kinase inhibitor, *mTORI* mTOR inhibitor

### FDG PET/CT assessment

**Fig. 2:**
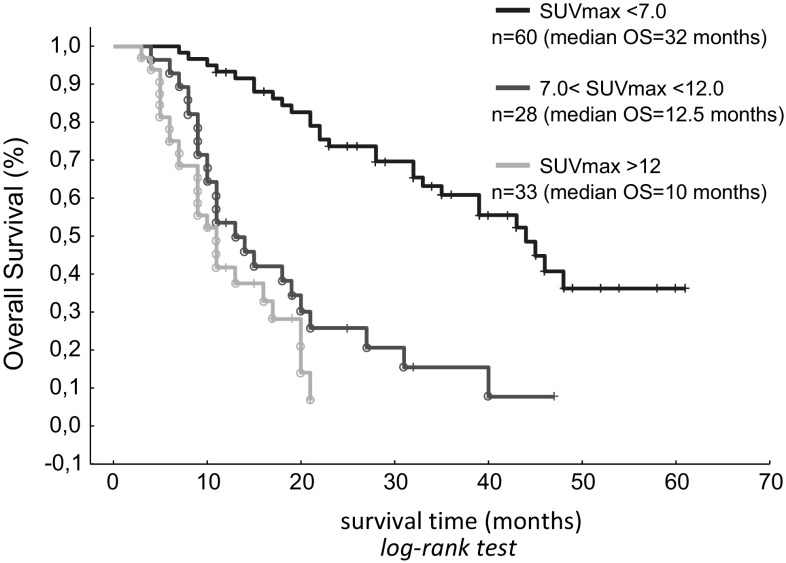
Overall survival curve of total 121 patients stratified by two cutoff points, max SUVmax 7.0 and 12.0

SUVmax measurements in all patients ranged from 1.3 to 30.0 (median 6.9). With SUVmax analyzed as a continuous variable, it was found that high SUVmax was associated with a shorter OS (Fig. 2; *p* < 0.003, hazard ratio 1.311, 95% CI 1.198–1.361). The impact of SUVmax on OS was compared with that of a range of standard risk factors. Multidimensional analysis of SUVmax in association with fitness status, calcium level and the gap between diagnosis and start of treatment (*p* < 0.016 in monodimensional analysis) revealed that SUVmax was a significant independent predictor of OS (Table 3).

**Table 3:** Univariate and multivariate Cox analyses of max SUVmax versus standard prognostic factors for advanced RCC

Risk Factor	Univariate Cox analyses			Multivariate Cox analyses		
	*P* value	HR	95% CI	*P* value	HR	95% CI
Max SUVmax (continuous variable)	< 0.003	1.311	1.198-1.361	< 0.002	1.277	1.163-1.389
Karnofsky performance status (< 80%)	0.022	2.107	1.121–4.133	0.311	0.593	0.243–1.623
Corrected calcium (> 10 mg/dl)	0.019	2.542	1.203–5.917	0.162	1.889	0.792–4.765
Hemoglobin (< lower limit of normal)	0.311	1.932	0.893–3.755			
Interval from initial diagnosis to treatment (< 1 year)	0.016	1.877	1.178–3.336	0.174	1.459	0.916–2.782
Age (> 65 years old)	0.522	0.883	0.514–1.373			
Sex (male or female)	0.760	1.114	0.545–1.976			
Pathology (clear or non-clear)	0.052	2.211	1.102–3.863	0.892	0.796	0.399–2.321

We first checked the correctness of selecting SUVmax = 7.0 as the cutoff value for establishing prognosis in a report [17] regarding a group of 101 patients. We categorized 121 patients from the current study into three subgroups according to their SUVmax, as suggested by that report [17]. In the current study, median OS for 60 RCC patients with SUVmax < 7.0 was 32 months, while in 28 RCC patients with 7.0 ≤ SUVmax < 12.0, median OS was 12.5 months (95% CI 4.97–19.45; *p* < 0.003).

For 33 patients (27%) with SUVmax ≥ 12.0, median OS was 10 months (95% CI 1.3–9.7). Differences in OS for these subgroups of patients were statistically significant (< 7.0 vs. ≥ 7.0 and < 12.0: *p* = 0.003; ≥ 7.0 and < 12.0 vs. ≥ 12.0: *p* = 0.04; Fig. 1). Regardless of the tumor size and the organs in which metastases were found, patients with a lower SUVmax had a longer OS than patients with a higher SUVmax.

## Discussion

We demonstrated that SUVmax in a FDG PET/CT examination is a useful prognostic marker of overall survival of patients with advanced RCC. Our results are consistent with those reported by Nakaigawa et al. (BMC Cancer (2016) 16:67) who showed that SUVmax was an independent prognostic factor for OS [17].

It is rational to claim that RCC with a high SUVmax would be associated with a poorer prognosis, because it is suggested that RCC with a fast metabolism requires more glucose as a source of energy.

Many researchers attempt to determine methods of establishing prognosis for RCC. The MSKCC classification recommended by Motzer et al. is the most common method [18] which divides patients into three subgroups: favorable-, intermediate- and high-risk patients with a median OS of 30, 14 and 5 months, respectively.

In our opinion, SUVmax could be a better prognostic marker compared to the risk factors used in the MSKCC if the results are confirmed and validated in future larger series of patients.

The main treatments in our study included those aimed at molecular targets that inhibited the biological activity of the tumor. As a result, the unique biological properties of RCC were significantly involved in obtaining clinical remission. Although the size of our study was relatively small, the results were statistically significant.

Some researchers suggest that FDG PET/CT is generally not appropriate for assessing RCC because of the urinary excretion of the radiopharmaceutical, which can mask the presence of primary lesions [19, 20]. Recently, several research groups have shown high usefulness of FDG PET/CT in assessing RCC response to molecular target-based treatments [21–23].

The prognostic assessment used in our study is clinically beneficial, since molecular target-based treatments, as opposed to the conventional cytotoxic antineoplastic agents, do not always cause an obvious response of the tumor. FDG PET/CT demonstrates that the uptake of FDG can be used not only as a prognostic marker before treatment, but also as a tool for a real-time assessment of the biological activity status of RCC.

## Conclusions

A preliminary SUVmax assessment conducted using FDG PET/CT can provide information useful in the prediction of survival of patients with advanced RCC.
